# Plasmonic Chirality Meets Reactivity: Challenges and Opportunities

**DOI:** 10.1021/acs.jpcc.4c08454

**Published:** 2025-02-06

**Authors:** Charlène Brissaud, Swareena Jain, Olivier Henrotte, Emilie Pouget, Matthias Pauly, Alberto Naldoni, Miguel Comesaña-Hermo

**Affiliations:** † Université Paris Cité, CNRS, ITODYS, 75006 Paris, France; ‡ Department of Chemistry and NIS Centre, University of Turin, Turin 10125, Italy; § Regional Centre of Advanced Technologies and Materials Department, Czech Advanced Technology and Research Institute, Palacký University Olomouc, Šlechtitelů 27, Olomouc 78371, Czech Republic; ∥ Nanoinstitut München, Fakultät für Physik, Ludwig-Maximilians-Universität München, Königinstraße 10, 80539 München, Germany; ⊥ Université of Bordeaux, CNRS, Bordeaux INP, CBMN, UMR 5248, F-33600 Pessac, France; # Université de Strasbourg, CNRS, Institut Charles Sadron UPR22, F-67000 Strasbourg, France; ∇ ENS de Lyon, CNRS, LCH, UMR 5182, F-69342 Lyon Cedex 07, France

## Abstract

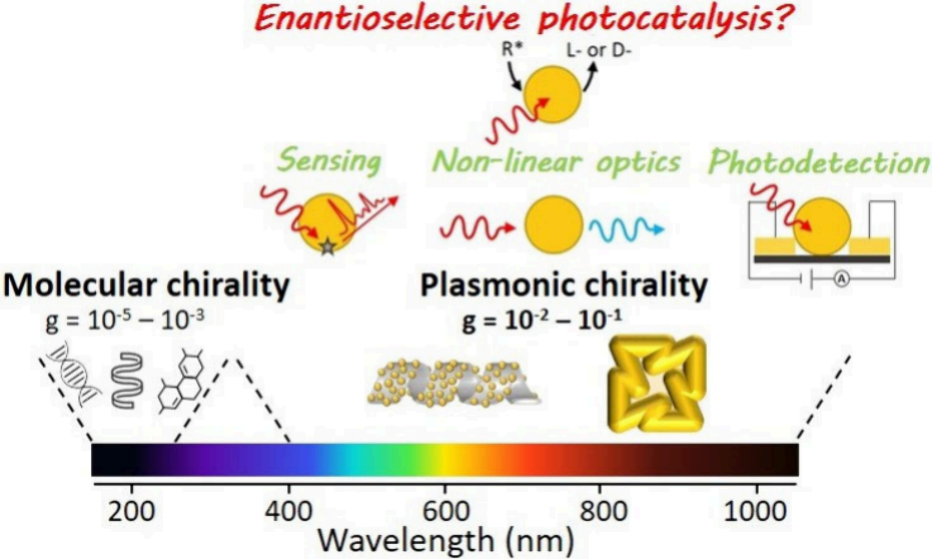

The unique optoelectronic features associated with plasmonic nanomaterials in a broad energy range of the electromagnetic spectrum have the potential to overcome the current limitations in the development of heterogeneous photocatalytic systems with enantioselective capabilities. Recent advancements in creating plasmonic structures with strong chiroptical features have already enabled asymmetric recognition of molecular substrates or even polarization-sensitive chemical reactivity under visible and near-infrared irradiation. Nevertheless, important developments need to be achieved to attain real enantioselective reactivity solely driven by plasmons. This Perspective discusses current trends in the formation of chiral plasmonic materials and their application as photocatalysts to achieve stereocontrol in photochemical reactions. We summarize the challenges in this field and offer insight into future opportunities that could enhance the effectiveness of these innovative systems.

## 1 Introduction

**Figure 1:**
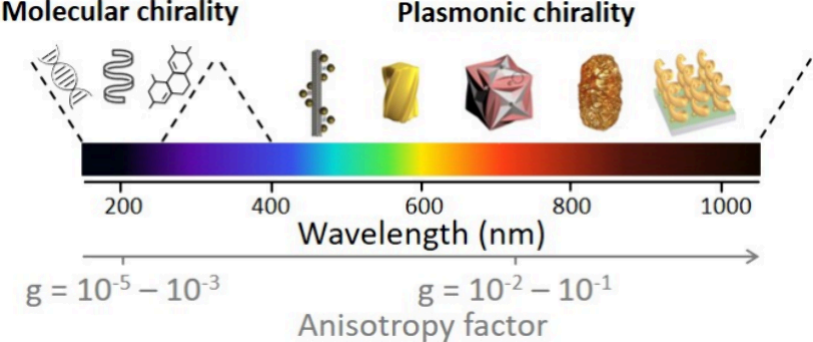
Comparison of the electromagnetic regions of chiroptical activity and anisotropy factors of common chiral molecular systems and different types of chiral plasmonic systems. 3D sketch of the plasmonic-DNA assembly was reproduced with permission from ref (5). Copyright 2012, Springer Nature. The twisted nanorod was reproduced with permission from ref (4). Copyright 2023, Wiley-VCH. The chiral nanocube was reproduced with permission from ref (2). Copyright 2018, Springer Nature. The wrinkled nanorod was reproduced with permission from ref (3). Copyright 2020, American Association for the Advancement of Science. The fully metallic helices were reproduced with permission from ref (10). Copyright 2009, American Association for the Advancement of Science.

In 1848, Louis Pasteur studied fermentation and distinguished two forms of tartaric acid by observing different responses to plane-polarized light.^1^ This led Lord Kelvin to coin the term “chirality” (1894), a word of Greek origin meaning “hand”. Indeed, hands are a classic example of chirality in nature, being composed by the same units but where one of them cannot be superposed to its mirror image. In general, this phenomenon can be broadly classified into structural chirality, exhibited by a pair of molecules that have both the same molecular formula and functional groups, while featuring a different structural configuration (i.e., enantiomers). As a result, enantiomers cannot be superposed. The different spatial orientation of molecular functional groups also causes optical chirality by altering their interaction with polarized electromagnetic radiation. Circularly polarized light (CPL) is generally employed to differentiate the interaction of a chiral entity with the handedness of light, either left- or right-handed (LCP and RCP, respectively), resulting in different extinction coefficients. Such phenomenon is called circular dichroism (CD) and can be defined as
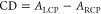
1where *A*_LCP_ and *A*_RCP_ are the absorptions for LCP and RCP light, respectively. Usually, the optical activity of chiral objects is measured through the dimensionless *g*-factor (also called anisotropy factor):
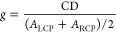
2The chiroptical properties of organic and biomolecules arise from their electronic and vibrational excitations, being usually weak and confined to the UV and a small fraction of the visible range of the electromagnetic spectrum. These characteristics limit their implementation in many applications. In this context, the use of plasmonic nanomaterials is of great relevance, given the strong extinction cross sections, intense electric field enhancements and broad range of responses associated with their localized surface plasmon resonances (LSPRs) in the visible and near-infrared (NIR). Moreover, such structures, particularly when fully inorganic, are more robust than organic molecules with respect to external changes such as pH, temperature or ionic strength, among others. In the last years, examples of chiral plasmonic nanostructures have been reported, either by synthesizing nanomaterials with chiral morphologies or by elaborating chiral assemblies of achiral nanoparticles (NPs).^2−5^ In both cases, these chiral plasmonic nanomaterials usually display chiroptical signals that are several orders of magnitude larger than those of molecular species, while spanning a much broader fraction of the electromagnetic spectrum (Figure 1). Therefore, chiral plasmonic structures have attracted increasing attention in the past years and have emerged as a new research direction in nanophotonics. Understanding the source of nanoscale chirality has been the focus of many studies and has led to the development of novel materials with potential applications in optoelectronics, catalysis, sensing or the formation of metamaterials, to name a few.^6−9^

Among the numerous applications of chiral materials, asymmetric (i.e., enantioselective) catalysis is particularly relevant, since it leads to the formation of enantiopure chemicals, being of paramount importance for the development of modern pharmacology and new formulations in the agrochemical and food industries.^11^ Usually performed in homogeneous solutions with molecular catalysts, important advances have been devoted to the development of asymmetric reactions in heterogeneous settings.^12^ Ideally, heterogeneous catalysts should offer the high activity and selectivity of their homogeneous counterparts while enabling improved separation and recycling, together with higher catalyst stability. Heterogeneous asymmetric catalysis relies mostly on two strategies: (i) immobilization of enantioselective molecular catalysts onto materials with high specific surface areas (>100 m^2^/g) such as mesoporous SiO_2_^13^ and (ii) functionalization of a (supported) metal catalyst with molecular modifiers, chiral molecules that render enantioselective the interaction between the catalytic surface and the desired molecule.^14^

Meanwhile, the field of visible light photocatalysis has experienced an important growth since the beginning of the 21st century, permitting the development of selective and more efficient chemical transformations through energy or electron transfer processes.^15^ Since solar radiation is an abundant, cheap and green source of energy, the development of photocatalytic approaches resonates today with the current need for more sustainable and affordable chemicals. Two main strategies in homogeneous catalysis have been developed recently in order to attain such goal.^16^ The first one consists in the use of a single bifunctional chiral photocatalyst. Here, the metallic center of an organometallic complex can work as the source of chirality and the catalytic/photoredox center.^17^ The second strategy consists in the implementation of a dual approach, in which two complementary catalysts with different functionalities (e.g., an organocatalyst and a photoredox catalyst) work synergically in order to attain enantioselective control.^18^ Despite such advancements, stereocontrol of photochemical transformations remains extremely challenging, given the short lifetimes and high reactivity characteristic of photogenerated intermediates. Accordingly, it would be interesting to combine the unique features of plasmonic photocatalysis with asymmetric reactivity, aiming at performing heterogeneous and asymmetric photocatalytic reactions driven by plasmons. In this vein, current efforts intend to leverage the unique optoelectronic features of plasmonic objects presented above while providing with asymmetric recognition of a given molecular transformation. The significant progress in the development of plasmonic systems with chiral attributes in recent years is a first step in this direction.

In this Perspective, we start by discussing current trends in the formation of chiral plasmonic materials, from the assembly of nonchiral plasmonic resonators into chiral structures to single objects with chiral morphological features. Subsequently, we present the impact of plasmonics in the chiral photogrowth of inorganic structures, polarization-sensitive organic reactivity or chiral recognition, while aiming as enantioselective photoreactivity driven solely by plasmons as main goal. Finally, we summarize bottlenecks encountered by the scientific community working in this field and propose our view on the opportunities that lie ahead.

## 2 Chiral Plasmonic Nanomaterials

Chiral plasmonic nanomaterials can be divided in two categories, depending on the origin of their chiroptical features. While the first one comprises nanoarchitectures made by the organization of achiral plasmonic NPs into chiral configurations, the second refers to the formation of intrinsically chiral plasmonic NPs through finely tuned nucleation and growth mechanisms in the presence of a chirality primer. The chiral induction derived from the interaction between a chiral molecule and an achiral plasmonic resonator, usually leading to lower chiroptical intensities than the previous two examples, has been left out of the present study for simplification purposes.

### 2a Assembly of Achiral Plasmonic Materials into Chiral Nanostructures

**Figure 2:**
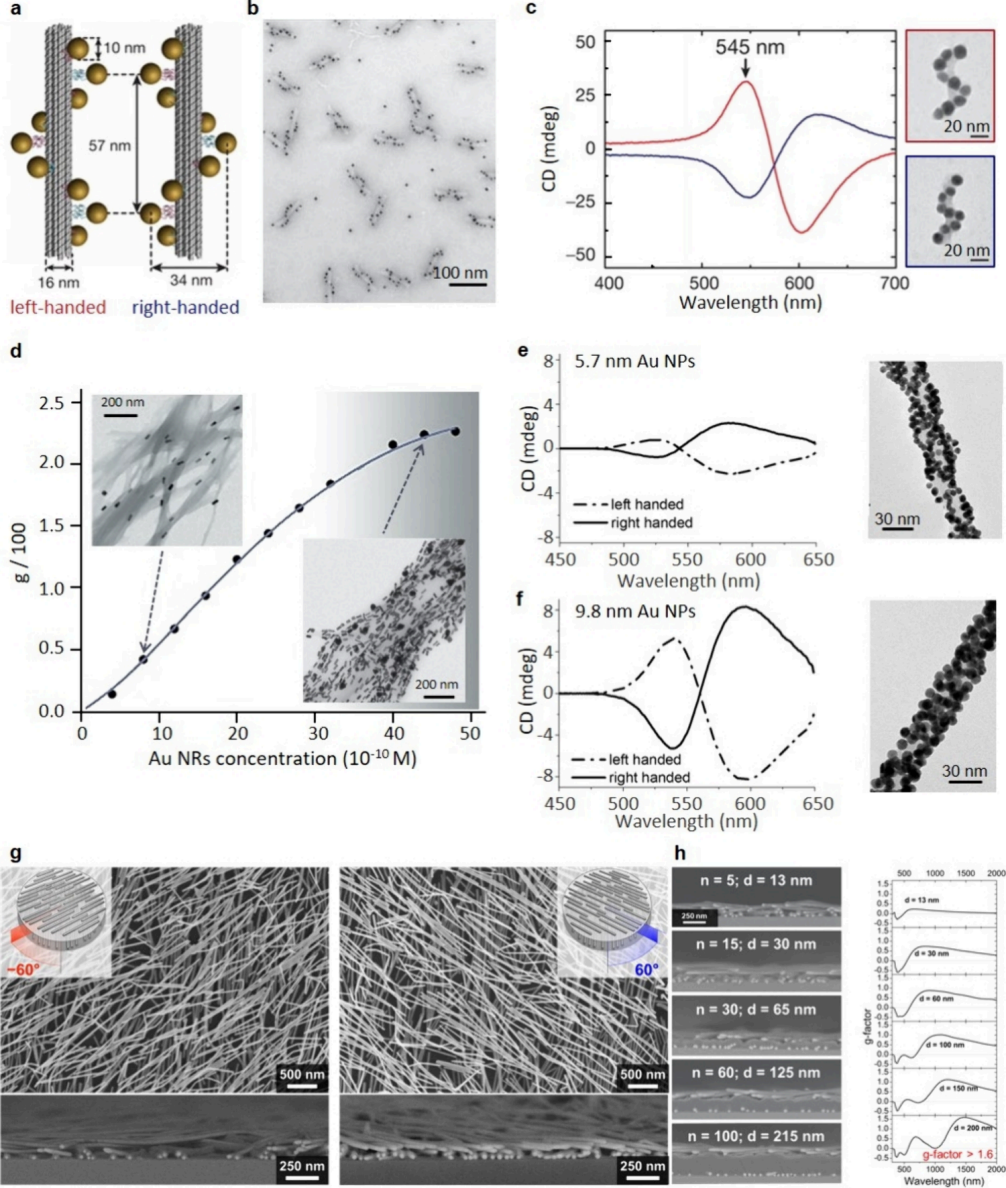
Illustrative examples of chiral assemblies. a, b) Schematic representation and TEM image of the DNA-based helical assembly of Au NPs. c) CD spectra of left- (red) and right-handed (blue) helices composed of 16 nm Au NPs. Reproduced with permission from ref (5). Copyright 2012, Springer Nature. d) Evolution of the *g*-factor of the Au-chiral polymer fiber nanocomposite with the concentration of Au NRs deposited. Reproduced with permission from ref (19). Copyright 2010, Wiley-VCH. e, f) CD spectra and TEM images of chiral silica nanohelices decorated with Au NPs of different sizes: 5.7 and 9.8 nm, respectively. Reproduced with permission from ref (22). Copyright 2017, American Chemical Society. g) Chiral metasurfaces formed by stacking 2 oriented nanowire layers with a director rotation between the layers. Reproduced with permission from ref (28). Copyright 2021, American Chemical Society. h) Cross-section SEM images of 2-layer chiral assemblies with a polyelectrolyte multilayer spacer of increasing thickness *d* (*n* is the number of polyelectrolyte layer pairs), which drastically changes the chiroptical properties of the assembly depicted in the right panel. Adapted with permission from ref (31). Copyright 2021, American Chemical Society.

Most of the time, chiral plasmonic assemblies are elaborated using chiral molecular templates such as DNA^5^ or organic fibers^19^ as colloidal scaffolds. For example, DNA origami are widely used templates for the fabrication of chiral nanostructures. In their pioneering work, Kuzyk and co-workers designed a DNA bundle containing 24 helices which offered 9 specific binding sites (Figure 2a,b).^5^ The addition of Au NPs coated with complementary single-stranded DNA allowed the authors to elaborate plasmonic helices with a CD signal close to the resonance wavelength of the individual Au NPs (around 550 nm), as depicted in Figure 2c. Remarkably, the authors showed the chiroptical response enhancement of the helices by coating each of the Au NPs with a silver shell of about 3 nm, resulting in a stronger coupling between the plasmonic NPs and leading to increased optical properties. In the same vein, Guerrero-Martínez and colleagues used the spontaneous assembly of Au nanorods (NRs) onto anthraquinone-based oxalamide fibers.^19^ The twisted shape of the organic filaments in solution induced a helical arrangement of the Au NRs with a preferential adsorption along their longitudinal direction. According to the authors, the observed chiroptical properties of such nanocomposites originate from the 3D chiral arrangement of the Au NRs on the substrate, leading to Coulomb interactions between the plasmonic NRs, as predicted by Fan and Govorov in the case of spherical Au NPs.^20^ By increasing the concentration of Au NRs onto the fibers, the authors were able to reach a maximum *g*-factor of 0.022 upon saturation of the surface (Figure 2d). Similarly, Song et al. used a peptide-based double helical scaffold to assemble Au NPs into long chiral chains with tunable parameters, such as interparticle distance, pitch of the helix and interhelical distance.^21^ Their plasmonic nanostructures display a strong chiroptical response in the visible range due to plasmonic coupling between the individual NPs. Depending on the aforementioned parameters, such peptide-based NP assembly could be used to prepare plasmonic nanostructures with tunable chiroptical properties. Another relevant example of chiral assemblies is the work of Cheng and colleagues on inorganic nanohelices.^22^ Using a sol–gel transcription procedure, the authors prepared nanometric silica helices starting from the self-assembly of a chiral gemini surfactant. Au NPs were then grafted onto the surface of the nanohelices, leading to a fully inorganic chiral plasmonic assembly with higher mechanical strength and improved stability in different solvents than those hybrids formed onto organic scaffolds. The influence of the size and arrangement of the Au NPs on the chiroptical features of the nanohelices were simulated based on a coupled dipole method. Increasing the diameter or the density of the Au NPs increased the CD signal of the nanohelices (Figure 2e,f). Moreover, grazing incidence spraying (GIS) allowed to tune the 2*D*/3D organization of such helices, confirming the impact of organization of the chiral objects on the optical properties as measured by Mueller matrix ellipsometry.^23^

Chirality can also be induced through the multilayer arrangement of achiral constituents into chiral structures. The simplest design consists of stacking two individual anisotropic NPs, such as NRs, on top of each other with a twist angle between them. Such system is particularly challenging to synthesize, given the numerous degrees of freedom present (e.g., twisting angle, lateral alignment and vertical distance between the layers). The resulting chiral dimer exhibits two optical modes whose resonance frequencies and amplitudes depend on the relative orientation of the NRs and the direction of the incident light, resulting in complex optical responses.^24^ Kuzyk and co-workers implemented the DNA origami strategy presented above for the formation of chiral Au NR dimers, leading to reconfigurable colloidal metamaterials through DNA strand displacement reactions.^25^ With a completely different approach, Yin et al. studied the optical properties of two orthogonally stacked Au NRs obtained by e-beam lithography.^26^ Their system displays two modes, the symmetric and the antisymmetric one, which are left- and right-handed, respectively. The authors showed that if the vertical distance between the NRs matches the resonance wavelength of the system, each mode is then excited solely by the polarization state of light that matches the handedness of the structure. In 2012, Zhao et al. showed that a multilayer stack of oriented Au NRs leads to the formation of circular polarizers in the visible and NIR wavelengths.^27^ Making such nanostructures (i.e., with dimensions in the range of tens of nm to be optically active in the visible range) by lithography is very challenging as it requires designing multiple layers by e-beam lithography separated by dielectric layers. Since this approach turns to be very costly and difficult to scale up to macroscopic devices, similar structures have been proposed using a bottom-up approach. For instance, chiral plasmonic metasurfaces consisting of a stack of oriented nanowires (NWs) or NRs have been produced by combining GIS and Layer-by-Layer (LbL) assembly.^28^ GIS relies on the low-angle spraying of 1D-NP suspension on a substrate, which allows forming monolayer thin films of Au NRs^29^ or Ag NWs^30^ in which all the objects are pointing in the same in-plane direction over areas greater than cm^2^. Such oriented monolayers can be stacked by LbL in a Bouligand structure in which the angle of orientation can be chosen independently in each new layer,^28^ which allows forming left- or right-handed helical multilayer superstructures (Figure 2g,h). It has been also shown that the chiroptical properties can be tuned over the entire UV, visible and NIR range by changing the angle and spacing between the oriented Ag NW layers, reaching *g*-factor values up to 1.6.^31^ To summarize, in these examples, the chiroptical properties of the assemblies arise from (i) a chiral transfer (from a chiral template to the assembly of achiral objects), (ii) a hybridization of the surface plasmon modes, which results in resonances that can couple differently with the states of incident CPL, leading to a different absorbance of LCP and RCP light at different wavelengths, or (iii) from the twisted stacking of linearly anisotropic layers.

### 2b Plasmonic Nanomaterials with Intrinsic Chiral Properties

**Figure 3:**
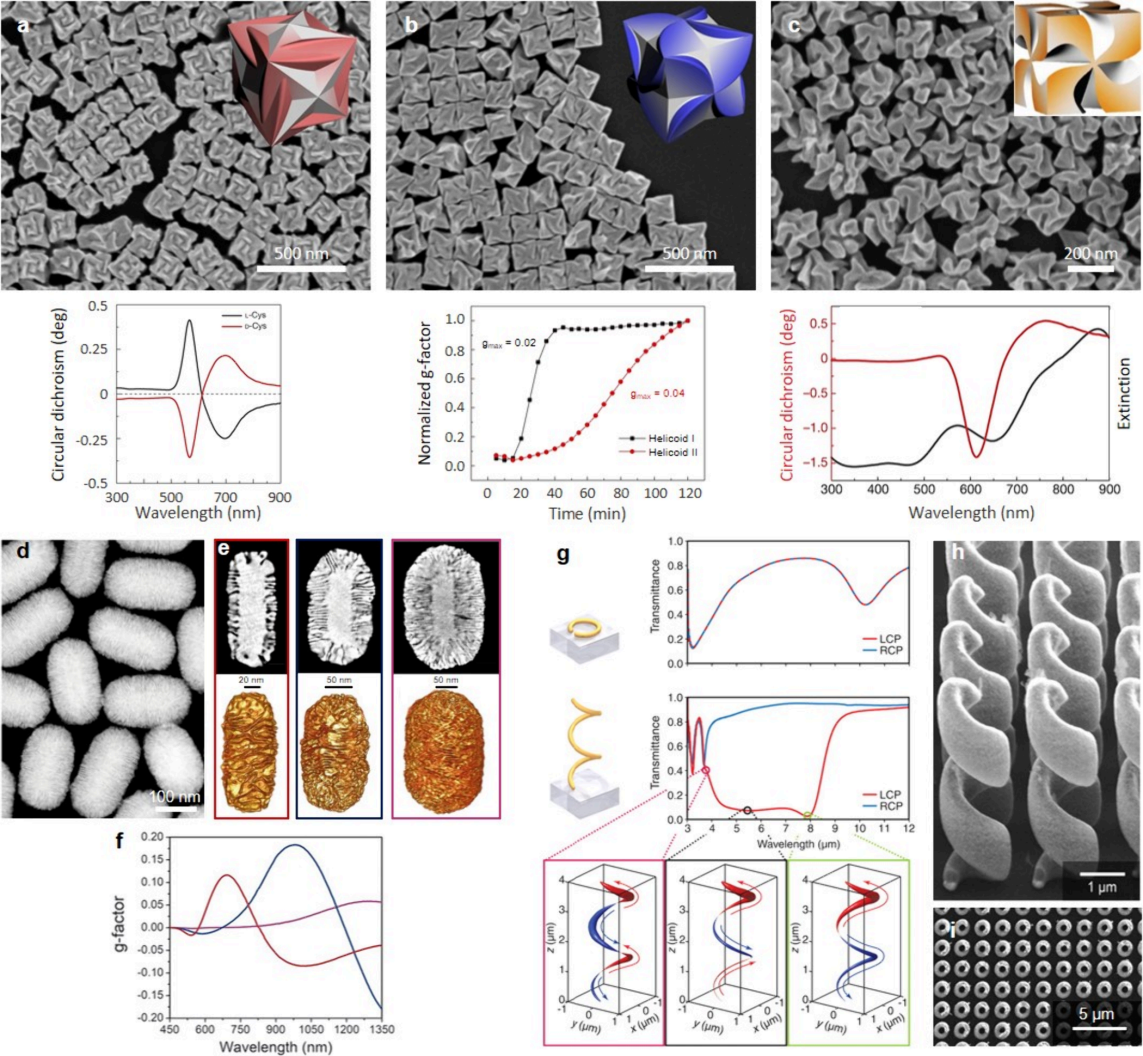
Illustrative examples of individual NPs with intrinsic chirality. a–c) SEM image of the helicoid I, II and III NPs (432 symmetry), obtained using cubic seeds and l-cysteine, cubic seeds and l-glutathione and octahedral seeds and l-glutathione, respectively. The insets show the chiral morphology of the different helicoids. The CD spectra of the helicoids I and III and the evolution of the *g*-factor of the helicoid II as a function of the reaction time are given below the corresponding TEM image. Reproduced with permission from ref (2). Copyright 2018, Springer Nature. d) HAADF-STEM image of anisotropic Au NPs with sharp chiral wrinkles. e) HAADF-STEM images of Au NPs of 165 × 73 nm, 210 × 112 nm and 270 × 175 nm (top, from left to right) together with the corresponding tomography reconstructions. f) Evolution of the chiroptical activity for NPs with increasing NPs size. Reproduced with permission from ref (3). Copyright 2020, American Association for the Advancement of Science. g) Normal incidence transmittance spectra of a planar split-ring resonator (top) and a left-handed metal helix (bottom) for an incident light coming from the air side. The snapshots illustrate the electric current distribution along the helix for three wavelengths and the absolute value of the current is represented by the thickness of the curve and the color code. SEM images of left-handed helices: h) oblique view and (i) top-view. Reproduced with permission from ref (10). Copyright 2009, American Association for the Advancement of Science.

The second category of chiral plasmonic nanosystems refers to individual nanomaterials that exhibit intrinsic morphology-related chiral properties at the single NP level. The synthesis of such materials typically relies on the use of chiral molecules to induce an asymmetric growth of the NP, often leading to twisted structures with exposed high-Miller-index facets and chiroptical properties.^2,4,32^ A well-known example of such chiral nanomaterials are the gold helicoids synthesized by Lee and co-workers.^2^ In this work, the authors used chiral amino-acids and peptides to imprint chirality during a seeded-growth process, using cubic and octahedral Au seeds. This method allows for the evolution of low-index-plane-exposed Au seeds into high-index-plane-exposed NPs. According to the authors, the chiral components interact enantioselectively with the surface of the NP, resulting in an asymmetric growth of the NP into helicoidal morphologies consisting of highly twisted chiral elements. Depending on the chiral molecule used as growth-directing agent and the morphology of the seeds, the authors obtained three different helicoids with chiral morphologies, one of them exhibiting a particularly high *g*-factor of 0.3 (Figure 3**a-c**). More recently, Ni et al. described the preparation of 4-fold twisted Au NRs using cysteine as a chiral inducer to direct the dissymmetric growth of single crystal Au NRs used as seeds.^4^ Through successive additions of the metal precursor, as well as precise control over the concentration of cysteine, growth temperature and size of the seeds, the authors were able to enhance both morphological and optical chirality of their materials, obtaining a maximum *g*-factor of 0.106. The surface energies of enantiomeric facets are different in the presence of adsorbed cysteine and this leads to an enantioselective growth of the chiral surface from the initial Au seeds in the form of ridge-like protrusions. González-Rubio and colleagues designed highly chiral Au NRs using a micelle-directed growth method in the presence of a chiral cosurfactant.^3^ According to their simulations, the cosurfactant molecules are responsible for inducing the assembly of the main surfactant in elongated worm-like micelles which tend to wind around the Au NRs used as seeds into helical structures. This adsorption promotes further growth of wrinkles at the surface of the Au NRs, resulting in the formation of NRs with chiral morphology and optical activity. By varying the concentration of seeds, the authors modulated the size of the chiral NRs and therefore the chiroptical properties of their materials, with *g*-factors ranging from 0.1 to 0.2 (Figure 3d–f). The spectacular progress in the colloidal synthesis of chiral Au NRs has been summarized recently in a perspective article.^33^ Top-down fabrication methods such as direct-laser writing, lithography techniques, or controlled etching have also been extensively studied for the elaboration of metallic chiral helices. For instance, Gansel and colleagues prepared three-dimensional micrometric Au helices arranged on a two-dimensional lattice using two-photon direct laser writing to design helical voids in a photoresist which were subsequently filled by electrochemical reduction of Au salt.^10^ The observed plasmon modes extend over the entire structure and are therefore strongly chiral themselves. Consequently, such arrays of plasmonic helices block LCP light (i.e., light with nonmatching handedness) while transmitting almost all incident RCP in the mid-IR range (Figure 3g–i).

It has been shown that CPL can also enhance the chirality of NPs synthesized in the presence of chiral molecules. Xu et al. used gold seeds with achiral shapes stabilized by achiral ligands, which were grown under CPL in the presence of different chiral dipeptides.^34^ The NPs obtained after illumination retained some similarity to the original achiral nanoprisms, but acquired out-of-plane protrusions resembling propeller blades, resulting in strong geometrical and optical asymmetry. Although the handedness of the objects is determined by their surface ligands, the maximum curvature of the blades is governed by the handedness of the CPL. Therefore, the chirality induced by the surface ligands can be enhanced or reduced depending on the handedness of the CPL used during the growth.

Averaging effects are unavoidable when measuring the optical properties of an ensemble of plasmonic colloidal NPs, leading to inhomogeneities related to variations in size and shape or the presence of structural defects, as well as possible interparticle interactions.^35^ In the particular case of colloidal populations of chiral plasmonic NPs, in which both enantiomers can coexist sometimes,^36^ averaging effects can lead to a misinterpretation of the chiroptical data. To overcome this issue, high resolution optical spectroscopy methods have been developed in order to characterize the chiroptical properties of single objects, even allowing to correlate their optical and structural properties when accompanied by electron microscopy techniques. For instance, circular differential scattering (CDS) spectroscopy has been developed by introducing CPL as the excitation source in a dark field scattering spectroscopy setup. Smith and co-workers used this technique to characterize chiral gold nanodumbbell dimers, given that the colloidal dispersions of these systems present a mixture of L-handed, R-handed and achiral systems, leading to a null chiroptical collective response.^37^ In this way, the authors were able to map the chiral properties of single dimers, which presented important chiroptical activities. More recently, a modified configuration of the same setup has been implemented in order to increase the detection limit of CDS, while allowing to remove artifacts from the characterization of chiral AuNRs that originated from the misalignment of the excitation light source.^38^

As we have just seen, chiral plasmonic nanostructures have been designed using many different approaches, allowing the elaboration of complex nanoarchitectures with extrinsic chirality due to interparticle interactions or the formation of NPs with intrinsic chiral features. These works exemplify the outstanding progress made in the field, leading to materials with unique and tunable polarization-dependent optical properties that are promising for numerous applications such as polarization-sensitive optical devices^39−41^ or chiral molecular sensing.^42^ Since each subcategory of systems exhibits chiroptical features derived from distinct origins (interparticle coupling versus chiral morphologies), their potential applications will inherently vary across different fields, thus highlighting the broad range of opportunities that lay ahead. In the next Sections of this Perspective, we will focus on the use of chiroplasmonic nanomaterials in polarization-sensitive catalysis and recognition together with their potential implementation toward enantioselective transformations. The recent advancements in colloidal chemistry toward the synthesis of anisotropic NPs with intrinsic chiral features showcased here will be particularly relevant in these domains. As will be discussed in more detail below, the potential exposure of superficial facets with chiral atomic ordering in these materials can induce an enantioselective interaction with molecular substrates. When combined with the optoelectronic properties of noble metal NPs, these morphological characteristics can produce synergistic effects, leading to enhanced chemical reactivity. However, precise characterization of the surface state of these materials, along with the stabilization of their metastable morphologies, remains a key challenge. We believe that the synthetic methods outlined here will be instrumental in advancing the emerging field of plasmon-powered asymmetric photocatalysis over the next decade.

## 3 Plasmonic Chirality Meets Reactivity

Plasmonic photocatalysis has been recently postulated as an interesting alternative to classical semiconductor-based heterogeneous photocatalysis, given the ability of plasmonic metals to produce more efficient chemical transformations utilizing a much larger fraction of the solar spectrum.^43−45^ As discussed before, the ability of certain nanometric metals such as Au, Ag or Cu to support intense LSPRs in the visible and NIR regions confers plasmonic NPs with unique optoelectronic features. Importantly, the large losses associated with the LSPR excitation of these materials allow the generation of nonthermalized (usually referred as “hot”) carriers. In this manner, highly energetic electrons and holes can be used to drive photoredox reactions. Hot carriers are particularly relevant since they offer a completely new landscape of chemical reactivity and selectivity that cannot be obtained with conventional thermal catalysis. The excited state formed between the photocatalyst and the substrate (i.e., the molecule to be transformed) upon photon absorption can follow a different reaction coordinate with respect to that of classical catalytic mechanisms.^44^ Accordingly, the evolution toward the formation of the products overcomes a new activation barrier that may be smaller than that corresponding to the reaction path available from the ground state (thermal pathway). This allows driving chemical processes that would remain inactive otherwise, leading to better efficiency.^46^ Moreover, the new reaction coordinate can lead to a completely new set of products, improving the selectivity of many photochemical reactions.^47^

### 3a Interaction between CPL and Plasmonic Materials

#### (1) Inorganic Growth

**Figure 4:**
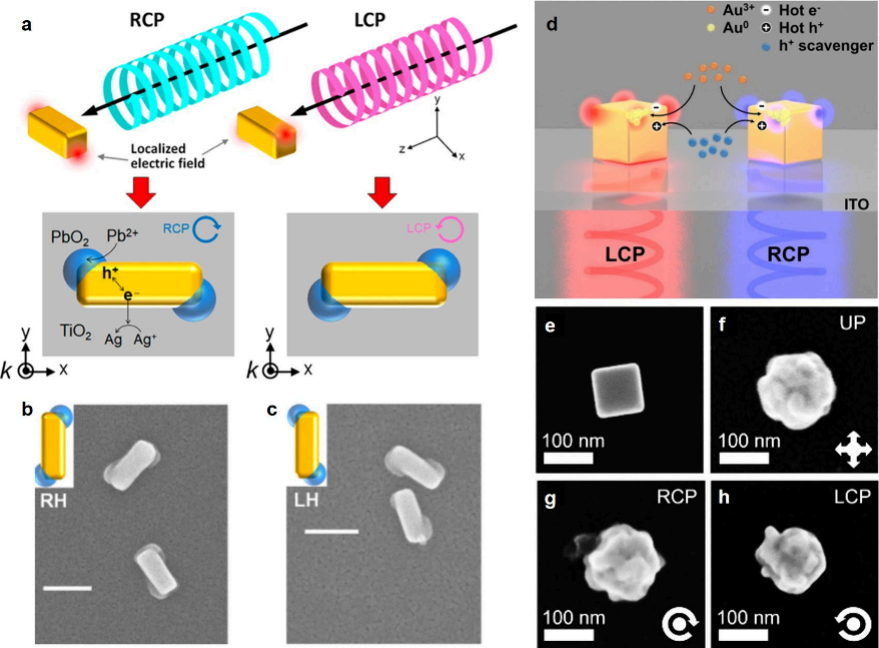
Illustrative examples of chiral inorganic growth induced by CPL. a) Scheme describing the interaction of CPL with an Au nanocuboid deposited on TiO_2_, leading to photoinduced charge separation and oxidation of Pb^2+^ to PbO_2_, which is selectively deposited on opposite corners. b, c) SEM images of the right- (b) and left-handed (c) nanostructures obtained upon irradiation with RCP and LCP light, respectively. Reproduced with permission from ref (48). Copyright 2018, American Chemical Society. d) Schematic illustration for the plasmon-induced chiral growth from an achiral Au nanocube on an ITO-coated glass slide, in which hot electrons reduce Au^3+^ to Au. e–h) SEM images of the Au nanocube before growth (e) and after growth under unpolarized light (f), RCP (g) and LCP (h) illumination. Reproduced with permission from ref (51). Copyright 2024, Wiley-VCH.

As described above, the growth of chiral inorganic single objects is usually achieved by using a chiral molecule as a chirality inducer during NP growth from nonchiral seeds. Another strategy relies on the use of CPL alone to induce the chiral shape. For instance, nonchiral gold nanocuboids have been adsorbed on a solid TiO_2_ thin film deposited on an ITO-coated glass plate and immersed in a solution of Pb^2+^ and Ag^+^ ions. When irradiated with CPL, nonthermalized charge carriers are generated, leading to the oxidation of Pb^2+^ to PbO_2_ and the reduction of Ag^+^ to Ag. This reaction preferentially occurs at sites where the electric field is the strongest (specifically, the corners of the cuboids), i.e. electromagnetic hot spots, resulting in the site-selective deposition of PbO_2_ (Figure 4a–c). Under RCP light, right-handed cuboids form predominantly, while left-handed cuboids are favored under LCP irradiation. Both enantiomers exhibit symmetrical CD spectra of moderate intensity in the visible range.^48^ The same synthesis strategy was later applied to the site-selective deposition of PbO_2_ on gold bipyramids.^49^ Ishida and co-workers developed all-silver chiral plasmonic nanostructures on a glass slide in a single step. This was achieved by irradiating right or left CPL through the glass into an aqueous solution of Ag^+^ and citrate ions, in the absence of NPs.^50^ The process involved the initial formation of achiral or racemic Ag NPs with anisotropic shapes due to photochemical electron transfer from citrate to Ag^+^, facilitated by CPL-induced electric fields. An achiral Ag nanoplate array was also transformed into chiral nanostructure arrays using hot electron reduction of Ag^+^ under CPL irradiation in the 600–700 nm range. Very recently, Lee et al. synthesized chiral Au NPs by irradiating Au nanocubes with CPL in the presence of Au^3+^, leading to chiral growth through the chiral distribution of hot electrons generated on the nanocube surfaces (Figure 4d–h).^51^ The study demonstrated that other metals could be photoreduced to form Au@Ag and Au@Pd (i.e., core@shell) chiral NPs. The optical properties of these structures were analyzed at the single-particle level using CD scattering, confirming their intrinsic chirality.

Recent studies have explored the potential of CPL to induce chiral shapes in objects dispersed in solution, moving beyond previous examples focused on achiral seeds on solid planar surfaces. Besteiro and colleagues developed an algorithmic model to simulate the growth of nanostructures influenced by the local excitation of hot electrons under CPL,^52^ reproducing successfully the chiral growth previously reported by Saito and co-workers.^48^ They also highlighted the challenges of achieving chiral NP growth in solution due to the averaging effects of random particle orientations relative to the light source. In a follow-up study, the same authors investigated how CPL could induce chiral patterns in initially achiral NPs using realistic plasmonic nanocrystal models.^53^ Their findings suggest that creating 3D chirality in solution via CPL is feasible through specific spatial distributions of near-field and hot electrons. They noted that anisotropic NPs with sharp edges are particularly effective for inducing chiral hot spots, although they observed that the 2D *g*-factors for NPs deposited on a planar surface were significantly higher than the 3D *g*-factors for those synthesized in solution. Furthermore, their model indicates that linearly polarized light (LPL) could induce chiral growth for NPs deposited on a planar surface but was ineffective in solution. Following these models, Ghalawat et al. compared the growth of chiral NPs under CPL in both solution and on a planar surface.^54^ Their experimental work demonstrated chiral symmetry breaking in Au@Ag nanobars in solution under CPL illumination, while the objects underwent partial galvanic replacement of the Ag shell by Au when immersed in an Au^3+^ solution. In contrast, when the same nanobars were immobilized on a solid surface and illuminated perpendicularly to their long axis, the asymmetry of their shapes increased. Another example of the synthesis of CPL-induced chiral NPs was recently published by Saito et al.^55^ Chiral plasmonic NPs were synthesized by depositing Ag on the surface of achiral Au NPs dispersed in solution. CD signals corresponding to the handedness of the irradiated CPL were observed with gold NRs or gold nanotriangles, whereas no CD signals were detected with gold nanospheres, in agreement with theoretical predictions.^53^

#### (2) Organic Transformations

Polarization-dependent transformations have been reported using chiral plasmonic NPs displaying different photocatalytic activities depending on the match/mismatch between CPL and the handedness of the plasmonic material. Hao and co-workers designed plasmonic resonators made of Au-gap-Ag NPs exhibiting chiroptical activity due to the presence of chiral cysteine in the nanobridged gap of the NPs.^56^ The photocatalytic activities of these materials were investigated through the reduction of 4-nitrophenol into 4-aminophenol under different irradiation sources. Interestingly, the authors found that the efficiency of the reaction depends strongly on the polarization of the incident light. Under LCP irradiation of their Au-gap-Ag with l-cysteine in the gap, the kinetic rate constant was estimated to be 5-fold, 10-fold and 12-fold higher than those obtained when the reaction is performed under linearly polarized light, RCP light, and in the dark, respectively. These results may be explained by the different generation rates of hot charge carriers in chiral plasmonic objects under irradiation with CPL, which has been predicted and observed in later studies.^57−60^ The polarization-dependent features of plasmonic hot carriers under CPL has been demonstrated both experimentally and theoretically using helical plasmonic arrangements as model photocatalysts.^61^ To do so, TiO_2_ NPs were added onto chiral silica nanohelices decorated with Au NPs (Figure 2e,f), leading to the formation of an inorganic nanostructure with chiroptical activity in the visible range. Such hybrid was used as photocatalyst to study the model photo-oxidation of an organic substrate under CPL. This study demonstrated experimentally that the transformation rate of the molecule was significantly enhanced when the polarization of the excitation light matched the handedness of the helices. The results were complemented with simulations which showed that the chiral arrangement of the plasmonic NPs onto the nanohelices induced a polarization-dependent interparticle coupling. Thus, when the NPs are excited with CPL matching the helicity of the hybrid, this coupling is maximized, leading to an enhancement of the electromagnetic field confinement and a higher density of photogenerated charge carriers. Such enhanced generation results in an improved photocatalytic activity. The above study illustrates the importance of supporting experimental work with theoretical calculations to better understand the mechanism underlying the polarization-dependent photoactivity of chiral plasmonic systems.

#### (3) Enantioselective Recognition

Enantioselective recognition poses a significant challenge in the fields of chemistry and biochemistry, particularly in the detection and production of enantiomers. The ability to selectively identify and differentiate between enantiomers is crucial due to the profound implications these compounds have in pharmacology, materials science, and environmental chemistry. Accordingly, adopting chiral structures is required to effectively recognize and produce the desired enantiomer.

**Figure 5:**
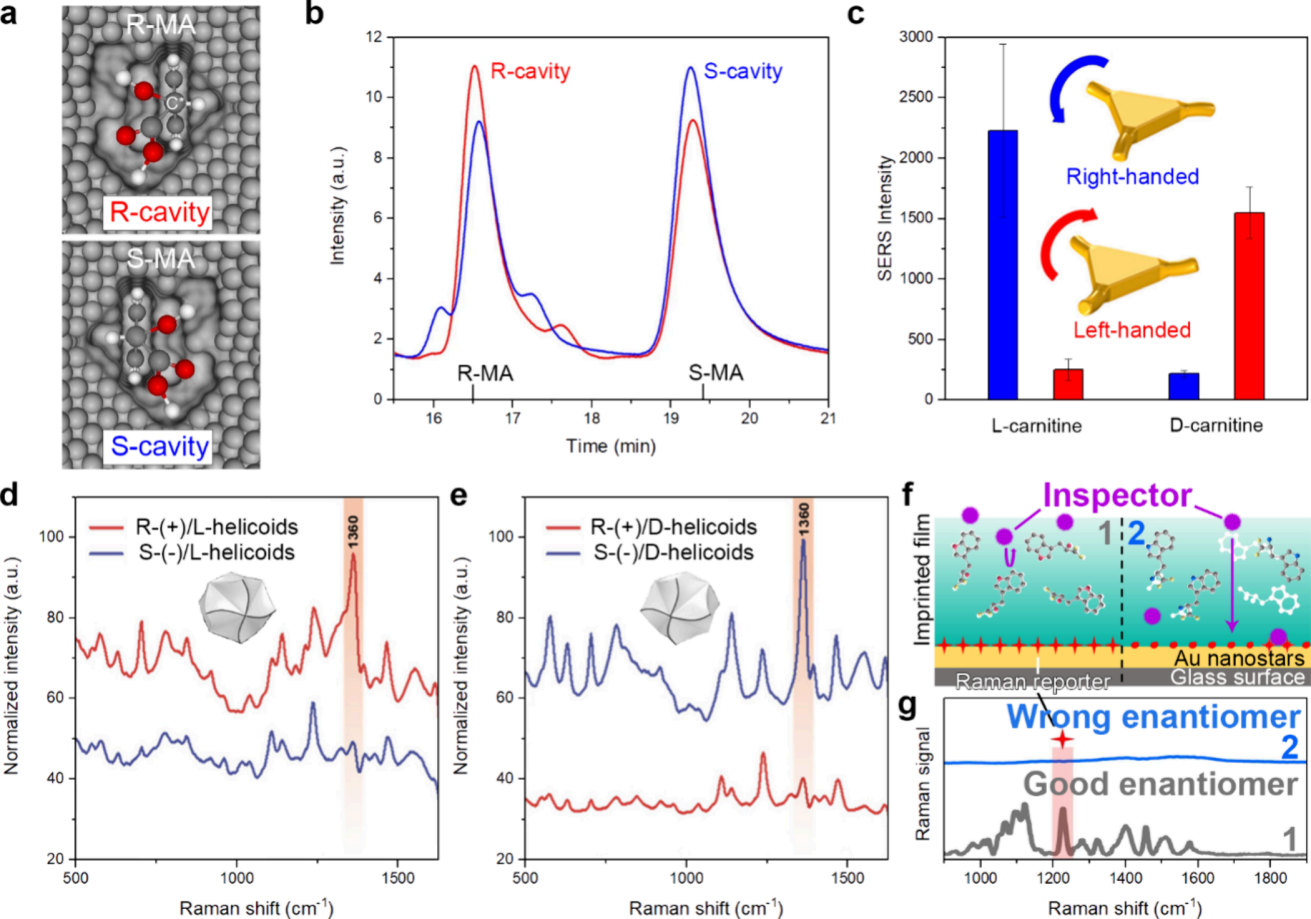
Illustrative examples of enantioselective recognition. a) Scheme depicting the R-cavity (top) and the S-cavity (bottom) imprinted in Pt films, and the corresponding mandelic acid enantiomer (R-MA or S-MA) sitting within the cavity. b) HPLC chromatograms of the electrosynthesis products obtained by an electrode imprinted with R-cavity (red) or S-cavity (blue). The retention times of R-MA and S-MA are 16.5 and 19.4 min, respectively. Reproduced with permission from ref (64). Copyright 2016, Springer Nature. c) SERS intensity obtained for l- and d-carnitine from right-handed (blue) and left-handed (red) Au propellers (depicted on the panel). Reproduced with permission from ref (67). Copyright 2020, American Chemical Society. d, e) Averaged SERS spectra of R- (red) or S-naproxen (blue) enantiomers on l- (d) or d-Au-helicoids (e). The corresponding Au-helicoids are depicted on the respective panel. Reproduced with permission from ref (70). Copyright 2024, American Chemical Society. f) Scheme describing the inspector recognition mechanism for SERS measurements on Au-nanostars in the case of the desired enantiomer (1) and a wrong enantiomer (2). The inspector degrades the Raman reporter if it diffuses through the chiral imprinted film. g) Raman spectra corresponding to the scenario depicted in (f) for the good enantiomer (gray) and the wrong enantiomer (blue). Reproduced with permission from ref (71). Copyright 2022, Springer Nature.

The first significant advancements in enantioselective recognition were demonstrated more than two decades ago by using chiral metal surfaces (i.e., engineered with specific kink sites).^62,63^ The enantiospecificity of the kink sites of the metallic surfaces was demonstrated with the electro-oxidation of d- and l-glucose, which showcased the crucial role of the local surface chemistry on enantioselective processes. Following these initial studies, researchers started to explore more complex designs. For instance, mesoporous platinum films imprinted with chiral molecules were used as chiral electrodes to induce the enantioselective synthesis of mandelic acid (MA) (Figure 5a).^64^ The authors performed the electrochemical reduction of the prochiral phenylglyoxylic acid using their Pt films encoded with a R-molecule and obtained R-MA in enantiomeric excess, and *vice versa* for their S-imprinted electrodes (Figure 5b). This evidenced that chiral modifiers on metallic electrode surfaces can enhance the electrochemical reactivity of one enantiomer while suppressing the other. Similarly, the enantioselective electrochemical production was performed on bimetallic films (Pt–Ir), highlighting a significant boost in durability and reproducibility over several cycles, compared to the monometallic film.^65^ Interestingly, the local electric field enhancement resulting from plasmonic NPs enables a highly sensitive detection of chiral molecules.^66^ Recent advances have leveraged this plasmonic effect to enhance the detection of enantiomers through surface-enhanced Raman spectroscopy (SERS).^67−70^ By using chiral plasmonic structures, researchers can achieve enantioselective recognition at lower concentrations than what was previously possible. Following this approach, Ma et al. synthesized left- and right-handed Au chiral propellers in the presence of l- or d-cysteine, respectively, which induced the desired anisotropy.^67^ These nanostructures were demonstrated to be excellent candidates for the SERS recognition of chiral biomolecules. While the SERS signal obtained for l-carnitine was 10 times higher on the right-handed Au propellers than the left-handed one, the intensity obtained for d-carnitine was 7 times lower on the right-handed Au propellers compared to the left-handed one (Figure 5c). More recently, Huang et al. synthesized Au nanostars and used them as a SERS-based inspector recognition mechanism, allowing the discrimination of various amino-acids, with enantiomeric excess that can be measured at ultratrace level.^69^ Similarly, Skvortsova et al. observed clear differences in SERS signals for naproxen, penicillamine or propranolol depending on the handedness of Au helicoids, highlighting the critical roles played by electric fields and optical chirality in chiral discrimination (Figure 5d,e).^70^ By coupling SERS with chiral imprinted cavities, Arabi et al. obtained an absolute recognition of specific enantiomers.^71^ This method consists in a two-steps process. First, the recognition of the chiral molecules occurs on the imprinted cavities (corresponding to the desired chiral molecule) of a polymer film through the immobilization of the correct enantiomer. Next, an inspector molecule is released in the solution. In presence of the desired molecule, the inspector is blocked by the filled cavities of the film. Otherwise, the inspector reaches the Raman reporter molecules present at the SERS substrate (Figure 5f). Subsequently, the inspector degrades the Raman reporter, hence suppressing the Raman signal (Figure 5g). This shows the advantages of combining plasmonic structures with chiral recognition, leading to innovative applications in sensing and detection at the molecular level. In the future, we expect that researchers will couple plasmonic effects with electrochemical reactions to harness solar energy for driving enantioselective processes, resulting in a more sustainable and efficient approach to chiral compound detection and production.

### 3b Asymmetric Photocatalysis Driven by Plasmons: Challenges and Opportunities

**Figure 6:**
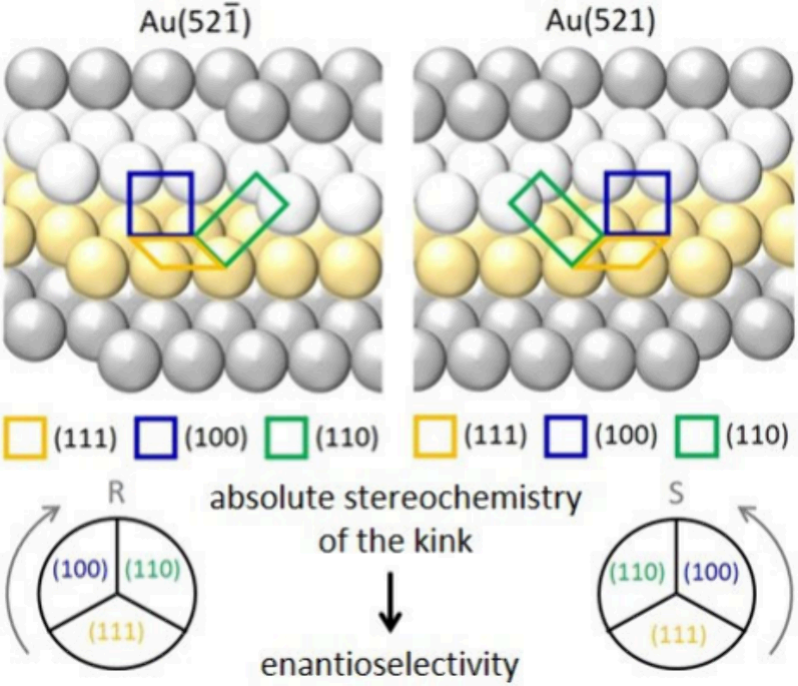
Schematic illustration of a chiral Au catalytic surface. The atoms constituting the surface are colored to highlight its ABC packing. Adapted with permission from refs (4) and (78). Copyright 1999, American Chemical Society, and 2023, Wiley-CH, respectively.

Most reports describing the interplay between chiral plasmonic systems and chemical reactivity are limited to polarization-dependent phototransformations (both, for organic and inorganic reactivities alike) or racemic differentiation reactions induced by plasmons. To the best of our knowledge, only very rare works can be found in the literature describing actual asymmetric photoreactivity in this context. For instance, Wei and colleagues showed that plasmonic metallic nanohelices can be used as chiral platforms to mediate the photoinduced formation of chiral products.^72^ To do so, a prochiral molecule, the 2-anthracenecarboxylic acid (2-AC), has been stereoselectively adsorbed on the surface of Ag nanohelices, forming enantiomorphous dimers that display opposite facial stacking (L-L or R-R) depending on the handedness of the plasmonic substrate. Upon irradiation with nonpolarized light, the cyclodimerization of the L-L and R-R stacking of 2-AC leads to the enantioselective formation of R- and L-dimers, respectively. Current bottlenecks impeding the appearance of other examples arise because the chiroptical responses of the different plasmonic nanomaterials presented above cannot, by themselves, ensure an asymmetric interaction with a given organic substrate. This is due to the large size mismatch between the wavelength of the electromagnetic field, the chiroptical features of the plasmonic objects or assemblies arising at the nano/microscale and the asymmetric character of a molecular species, appearing at the atomic level. As a consequence, a molecule adsorbed at the surface of a plasmonic nanostructure may remain inactive with respect to the potential chiroptical properties of the latter, given that their origin is the result of light-matter interactions at much larger size scales. In order to overcome such mismatch, the plasmonic object must exhibit chirality at the atomic level to achieve an enantioselective interaction with a given molecule. Such chirality can emerge on a crystal surface if the atomic arrangement exposed lacks any mirror-symmetry perpendicular to the surface.^73^ A strategy to achieve this effect relies on the synthesis of nanostructures with exposed high-Miller-index facets. The presence of defects on such surfaces results in the formation of kink sites, where terraces and smaller facets exhibit a distinct arrangement that leads to a specific handedness (Figure 6). These intrinsically chiral surfaces are more likely to interact preferentially with an enantiomer of the same handedness and are thus more prone to catalyzing chemical transformations enantioselectively. Among the plasmonic chiral structures already discussed in this Perspective, those with intrinsic chiral morphology present, in many cases, high-Miller-index facets.^2,4^ In this line, Bainova and co-workers presented recently the use of chiral (L- and R-) Au NPs for the plasmon-induced homolysis of a C–O–N bond of an alkoxyamine substrate presenting chiroptical activity in the visible range.^7^ The experiments showed that only when the helicity of the substrate, the polarization of CPL and the chiroptical activity of the substrate matched, the reactivity was maximized. The authors discussed that the importance of the latter parameter could not be explained as a difference in the surface adsorption capabilities of the molecule onto the metallic surface. Instead, they proposed that a plasmon-induced resonant energy transfer between the metal NP and the alkoxyamine leads to an electronic transition in the latter that can be sensitive to both the chirality of the object and the optical activity of the substrate.

As mentioned before, we believe that plasmonic objects with intrinsic chiroptical activities and exposing high-Miller index facets represent the best and simplest opportunity in order to move the field toward photoinduced asymmetric reactivity. Unfortunately, in many cases these geometries are particularly unstable, given that they expose high energetic surfaces and are therefore prone to fast reshaping. A recent report discussed the improved structural stability of chiral Au NRs exposing such highly energetic facets through their coating with different materials, such as mesoporous silica or organic polymers.^74^ This could be a promising strategy toward the formation of more stable heterogeneous asymmetric photocatalysts provided that the coating layer does not impede access to the metal surface. The formation of a mesoporous silica shell can be particularly interesting, given its sieving effect toward larger molecules that can lead to an improvement in the selectivity of the final catalyst. Similarly, formation of hybrid materials in which the chiral plasmonic object is coated with a metal–organic framework (MOF) presents important advantages for the development of sensors, theranostic tools or heterogeneous reactors.^75^ Indeed, the improved structural stability^76^ and the formation of enantioselective catalytic pockets that can be created within the porous structure^77^ are important assets that, when combined with the generation of nonthermalized charge carriers and high electromagnetic field enhancements within the metallic core, can lead to the development of a new generation of asymmetric photocatalysts.

Finally, a novel emerging strategy for enantioselective chemistry driven by chiral nanostructures is polaritonic chemistry,^79^ in which highly confined electromagnetic fields interact strongly with molecular vibrational or electronic modes to create hybrid light-matter states known as polaritons. These polaritons exhibit distinct reactive properties compared to their uncoupled counterparts, offering new selective routes in synthesis.^80^ Cavities capable of selectively confining electromagnetic fields of a specific chirality could pave the way for chiral polaritonic chemistry,^81^ facilitating the selective synthesis of specific chiral molecules. Currently, most research on chiral cavities is theoretical,^82,83^ since experimental implementation of such structures requires chiral mirrors that preserve the helicity of light upon reflection. The first demonstration of chiral reflectivity in 2015 was limited to the microwave region,^84^ later extended to the infrared^85^ and visible^86^ ranges using lithography, or more recently by a bottom-up approach.^87^

## Conclusion

Plasmonic photocatalysis represents a unique opportunity to photomodulate the enantioselective character of different chemical transformations given the extremely high extinction cross sections and chiroptical asymmetric factors associated with such materials. Recent advancements in colloidal chemistry have enabled the synthesis of plasmonic NPs and assemblies with outstanding chiroptical features across a large portion of the solar spectrum. These developments have expanded the potential applications of chiral plasmonic systems, facilitating their implementation in polarization-sensitive reactivity and asymmetric chemical sensing. In this vein, the ability to harness solar energy for asymmetric synthesis represents a major step forward in catalysis. However, further work is needed in understanding the atomic-scale interactions between plasmonic materials and molecular substrates. Achieving precise control over these interactions is essential for optimizing catalytic efficiency and reaction selectivity. Moreover, gaining mechanistic insights into the physical processes involved and elucidating the chemical intermediates formed during these reactions are critical to advancing the field. Prior to that, a comprehensive understanding of the optoelectronic and structural features of these novel and exotic materials still needs to be attained. The use of advanced spectroscopy and electron microscopy techniques are fundamental tools in order to achieve these goals. For instance, some authors have discussed that future advancements in electron tomography could permit to map locally the atomic handedness of the exposed crystalline facets of NPs, leading to a major leap in the comprehension of the structural properties responsible for the chirality of these objects.^33^

The promise of plasmon-induced asymmetric reactivity lies not only in its fundamental scientific interest but also in its potential applications within the pharmaceutical and agrochemical industries. With continued advancements, plasmonic photocatalysis could provide a sustainable and cost-effective alternative to current synthetic methods that are heavily reliant on fossil fuels. This could contribute significantly to reducing the environmental footprint of chemical manufacturing, while offering new pathways for the efficient synthesis of chiral compounds. Moreover, the ability of hot charge carriers and electromagnetic field enhancements inherent to plasmonic excitation to modulate chemical reactivity can be of particular interest to open new synthetic avenues that are not available today. As the field progresses, the integration of plasmonic photocatalysis into practical applications holds the potential to revolutionize both green chemistry and industrial practices in the coming years.
